# Safe injection awareness and practices among nursing staff in an Egyptian and a Saudi hospital

**DOI:** 10.1186/s42506-019-0018-5

**Published:** 2019-07-03

**Authors:** Manal M. Anwar, Alshimaa A. Mohamed Lotfy, Afaf A. Alrashidy

**Affiliations:** 1 Department of Public Health and Community Medicine, Faculty of Medicine, Beni-Suef University, Beni Suef, Egypt; 2 Ministry of Health, Al Qassim Region, Kingdom of Saudi Arabia

**Keywords:** Awareness, Practice, Safe injection, Nurses

## Abstract

**Background:**

Unsafe injection practices are an occupational hazard among the nursing staff. Awareness of nurses’ staff members about safe injection practices may vary between different hospitals according to the policies adopted for staff training and systematic auditing.

**Aim:**

To assess awareness and practice of safe injection among nursing staff in a Maternal and Child Hospital, Qassim Region, Saudi Arabia, and Beni-Suef University Hospital, Egypt.

**Methods:**

A cross-sectional study using a structured questionnaire. Observations included 500 injections (250 from each hospital) from October to December 2017.

**Results:**

The mean awareness total scores in both hospitals were 9.98 ± 1.76 and 11.12 ± 0.96 respectively with a significant difference among observed nurses (*P* = 0.001). The mean safe injection practice total score was 27.13 ± 3.11 and 27.39 ± 2.17. Past year safe injection training was received for 95% and 70% for observed Egyptian and Saudi group of nurses. The majority of nurses (98.8%) were aware of the importance of safe injection practices to minimize blood-borne diseases, and 95.2% of them were aware of the placement of sharps disposal box beside the place of injection procedure. Exposure to the past year needlestick injuries (NSIs) was higher among the observed Egyptian nurses (*P* = 0.001). Using appropriately stored and refilled disinfectants was done by 48.5% and 51.5% of the observed nurses. Needle separation from its syringe inside sharps disposal box and sharps disposal boxes near patient care areas were observed in 95.2% and 95.6% of both hospitals respectively.

**Conclusions and recommendation:**

Nurses of both hospitals have good awareness and practice of injection safety. This might be attributed to the adoption of appropriate training courses. There is a need for continuous training sessions and auditing for nursing staff to ensure safe injection practices.

## Introduction

Unsafe injection practices by healthcare providers pose a risk for patients and healthcare workers especially infectious and non-infectious adverse events and often are associated with a variety of improper procedures and unsafe settings.

Safe injection practices are a set of preventive measures aiming to optimally perform a safe patients’ injection manner; a “safe injection” should not harm the recipient, should avoid exposing the health care provider to any potential risk, and should not result in a hazardous waste for the community [1].

Four main potential risks might pose a direct patient hazard, namely *re-use of injection equipment*, where administration of about 16 billion injections are encountered worldwide with about 40% of which involves re-use of injection equipment [2]; *accidental needlestick injuries* (*NSIs*) for health care provider resulting in a total of 3 million accidental NSIs in a WHO survey [3]; *overuse of injections*, where various surveys conducted in different settings indicated a high (up to 56%) at least one injectable preparation was prescribed with an annual ratio of 1.7 to 11.3 injections per person per year [4]; and unsafe sharps waste disposal, where inappropriate collection and disposal of sharp wastes put the health care practitioner and the waste handler including the community at risk of sharps injuries with consequential blood-borne infections [5].

Safe injection practice is a component of standard precautions aimed at maintaining basic levels of patient safety and provider protections, which necessitates proper administration of an injection by a well-trained qualified healthcare provider under complete aseptic technique using a sterile device (syringe, needle, etc.) with its appropriate disposal in a puncture-proof sharps discard container [6, 7].

Proper injection equipment disposal is required not only to check the reuse of disposable syringes but also to minimize the opportunities of avoidable healthcare workers’ (HCWs) potential risks [8].

Improper syringe disposal and NSI incidents reflect that there is a wide gap between healthcare provider knowledge and their practice; therefore, injection-related behavioral changes are required that might be brought by continuous training and regular audit and supervision. After HCWs’ training as regards injection safety and waste management, behavior change and needle recapping were significantly reduced, and safety box use was increased [9].

Unsafe injection practices add to the global burden of blood-borne disease. This accounted for 14% of HIV, 25% HBV, 8% HCV, and 5% of bacterial infections worldwide and for 28 million preventable disabilities [10].

Lack of implementations of guidelines for safe injection practices and medical waste management may result in unsafe injection for healthcare providers as well as patients’ blood-borne risks. The aim of this study was to assess safe injection awareness and practices among nursing staff in an Egyptian and a Saudi hospital.

## Materials and methods

### Study design

The study design is a cross-sectional comparable design.

### Study setting

The study was conducted in Beni-Suef University Hospital (BSUH), Beni-Suef, Egypt, and Maternal and Child Hospital (MCH), Qassim, Saudi Arabia, in the period from October to December 2017. The two settings were comparable where the study population included nursing staff of intensive care units, emergency departments, and inpatient wards.

### Sample size

A convenient sample of 500 nurses was included in this study. A total of 258 nurses offering direct patient care out of 375 nurses accepted to participate from BSUH; 250 of them continued their participation in the study and 8 withdrew. As for the participating nurses from MCH, 250 out of 453 nurses participated in the study.

### Sampling selection method

We tried hard to make the total population as we included all nurses who offered injection (258/375 were selected from the BSUH in Egypt and 250/453 were selected from the MCH-Qassim in Saudi Arabia).

During the study period, a single observation was carried out by the research team who visited the clinical departments of the hospital to observe injection practices of the nurses; while on duty, a subsequent personal interview was carried out to fill out a questionnaire aiming to assess their awareness as regards safe injection practices. A total of 500 injections were observed (250 from each hospital). A pilot study was done for 30 nurses in each of the selected hospital before the data collection to ensure the clarity and easy handling of the questions. Content validity was assessed by reviewing the literature. The reliability of the questionnaire was calculated using the test-retest method, and no statistical differences were found.

### Tools of study

A structured questionnaire divided into 3 parts was used to assess awareness, practices of safe injection, and accidental NSIs and sharps disposal as follows:

- Part 1: A 12-item questions to assess their awareness about injection safety. The score for each question was zero or one. Questions with a correct answer were assigned a score of one point and incorrect answers questions were assigned a score of zero point. The total score was classified as: Poor (zero to five), fair (six to eight), and good (nine to 12).
- Part 2: A 31-item questions to explore nurses' reported practices concerning safety injection. The total score for this set of items ranged from zero to 31. Items that were correctly performed by the nurse were given a score of one point and observations of incorrect practices were given a score of zero point. The total score was classified as: poor (zero to 10), fair (11–20), and good (21–31). The tool used for observing nurses practices was as per World Health Organization (WHO) tool for the assessment of injection safety with some modifications.
- Part 3: History of accidental NSIs and sharps disposal for observed study nurses was assessed by four item questions.

### Statistical analysis

Data collected were coded and analyzed by the Statistical Package for Social Science (SPSS) version 20 (IBM, USA). Frequency distribution, percentage, and descriptive statistics including mean ± SD were calculated. Chi-square test was performed when indicated. *P* values of ≤ 0.05 were considered significant.

## Results

A total of 500 nurse practitioners from the two hospitals participated in the study. For the BSUH participants, the mean nurse age was 33.51 ± 10.17 years, 89% were females, 62% were married, the mean years of experience was 9.8 ± 7.7, and 63.5% of them had a bachelor level of nursing education. The mean age of MCH-Qassim nurses was 32.47 ± 8.53 years, 99% of them were females, 87% of them were married with a mean year of experience of 9.2 ± 8.4, and 80.3% of them had a bachelor level of nursing education.

### Methods of injections

Intravenous administration of medication was the commonest (42%) method of injection, followed by intravenous (IV) sample withdrawal (23.2%), intramuscular route (17.6%), and IV cannula insertion (17.2%). The most common method used for skin site disinfection before the injection was the single-use alcohol swab (87.4%), followed by cotton piece soaked by alcohol (12.3%), and only 0.2% used a cotton piece soaked with Betadine (Table 1).

**Table 1:** Methods of injection and skin disinfection among nurses in Beni-Suef, Egypt, and Maternal and Child Hospital (MCH), Qassim, Saudi Arabia, 2017

Items	Qassim Hospital (*n* = 250)	Beni-Suef UH (*n* = 250)	Total
	*N* (%)	*N* (%)	*N* (%)
Methods of injection			
IM	33 (13.2)	55 (22)	88 (17.6)
IV	171 (68.4)	39 (15.6)	210 (42)
Cannula	21 (8.4)	65 (26)	86 (17.2)
Sample	25 (10)	91 (36.4)	116 (23.2)
Methods of skin disinfection			
Single use alcohol swab	223 (89.2)	215 (86)	438 (87.6)
Piece of cotton soaked with alcohol	26 (10.4)	35 (14)	61 (12.2)
Piece of cotton soaked with betadine	1 (0.4)	0 (0)	1 (0.2)

#### Awareness about safe injection (12 items)

There was a statistically significant difference in favor of better awareness for 8 items including 2 items showing better training for safe injection practice and waste sorting among BSUH nursing staff members compared to only 3 items in favor for MCH-Qassim nursing members. Only the item shortage of syringe stock at any time during the past month showed no statistical significance (Table 2).

**Table 2:** Awareness of safety injection among the nurses in Beni-Suef, Egypt, and Qassim, KSA, 2017

Part I—12 items	Correct answer			*P* value
	Qassim Hospital *N* (%)	Beni-Suef UH *N* (%)	Total	
Is there an implemented safe injection policy and procedure in your hospital?	233 (93.2)	245 (98)	478 (95.5)	0.001*
Are there implemented infection control policies and procedures in your hospital?	241(96.4)	248 (99.2)	489 (97.8)	0.001*
Are there implemented policies and procedures for post-exposure needlestick injuries?	234 (93.6)	244 (97.6)	478 (95.6)	0.001*
Have you received any training for safe injection during the past year in your hospital?	176 (70.4)	237 (94.8)	413 (82.6)	0.001*
Do you know about measures to be taken after NSI?	240 (96)	232(92.8)	472(94.4)	0.001*
Are sharps disposal box present beside the place of injection procedure?	228 (91.2)	248(99.2)	476(95.2)	0.001*
R-During the past month, was there a shortage of sharps disposal box?	201 (80.4)	180 (72)	381(76.2)	0.018*
R-Do you have shortage of syringes stock at any time during the previous month?	186 (74.4)	187(74.8)	373(74.6)	0.500
Are there implemented policies and procedures for waste sorting?	226 (90.4)	246(98.4)	472(94.4)	0.001*
Have you received training for waste sorting?	175 (70.0)	212(84.8)	387(77.4)	0.001*
Do you know about blood-borne diseases?	250(100)	244(97.6)	494(98.8)	0.030*
Have you received 3 doses of HBV vaccination?	156(62.4)	190 (76)	346(69.2)	0.001*

*R* items that are negatively worded and the correct response is NO

*P value ≤ 0.05 is considered significant

The comparison of the nurses in the two hospitals as regards the safe injection awareness score showed a higher mean total score of 11.12 ± 0.96 for BSUH compared to that of 9.98 ± 1.76 for MCH-Qassim (*P* < 0.001) (Table 3).

**Table 3:** Comparison of safe injection awareness score among the nurses in Beni-Suef, Egypt, and Qassim, KSA, 2017

Score	Hospital			
	Qassim Hospital *N* (%)	Beni-Suef UH *N* (%)	Total *N* (%)	*P* value
Poor awareness (score 0–5)	5 (2.00)	0 (0.00)	5 (1.00)	0.001*
Fair awareness (score 6–8)	40 (16.0)	2 (0.8)	42 (8.4))	
Good awareness (score 9–12)	205 (82.0)	248 (99.2)	453 (90.6)	
Mean ± SD of the total score (total score = 12)	9.98 ± 1.76	11.12 ± 0.96	10.53 ± 160	

*P value ≤ 0.05 is considered significant

#### Safe injection practices (31 items)

There was a statistically significant difference in favor of better safe injection practice in 6 items for BSUH and MCH-Qassim nursing staff each. The observation of BSUH nursing staff members showed a higher percentage for intact needles mounted on its syringes inside sharps disposal box (97.6% vs 92.8%) for MCH-Qassim nursing members while for the item “washing hand or using alcohol hand rub before wearing gloves”, 78% and 92% of MCH-Qassim and BSUH nursing staff were observed to perform a correct hand hygiene practice (Table 4).

**Table 4:** Safe injection practices among the nurses in Beni-Suef, Egypt, and Qassim, KSA, 2017

Part II—31 items	Observations			*P* value
	Qassim Hospital *N* (%)	Beni-Suef UH *N* (%)	Total	
There are posters/handouts for safety injection	181 (72.4)	205 (82.0)	386 (77.2)	0.007*
R-There are needles outside its sheath or not disposed	192 (76.8)	204 (81.6)	396 (79.2)	0.225
R-Is there any needle separated from its syringe inside sharps disposal box?	232 (92.8)	244 (97.6)	476 (95.2)	0.008*
Is there any sharps disposal box near patient care areas?	234 (93.6)	244 (97.6)	478 (95.6)	0.001*
R-Are needles recapped after the injection?	218 (87.2)	218 (87.2)	436 (87.2)	0.553
R-Reusing the syringe between the patients	242 (96.8)	243 (97.2)	485 (97.0)	0.500
R-Keeping the syringe to reuse for the same patient again	245 (98)	243 (97.2)	488 (97.6)	0.772
R-Are sharps disposal boxes overfilled by more 3/4 of their sizes?	227 (90.8)	227 (90.8)	454 (90.8)	0.500
R-Are there any sharps used and not disposed in sharps disposal boxes?	221 (88.4)	220 (88)	441 (88.2)	0.501
After injection, are syringes or needles disposed off immediately?	219 (87.6)	215 (86)	434(86.8)	0.692
Does the injector use a new closed syringe every time before injection?	238 (95.2)	240 (96)	478 (95.6)	0.828
Does the injector prepare safety injection procedure on clean table or tray before injection?	242 (96.8)	241 (96.4)	483 (96.6)	0.500
Does the injector wash his hand routinely before intravenous injection?	217 (86.8)	216 (86.4)	433 (86.6)	0.500
Does the injector wear new gloves before intravenous injection?	235 (94)	228 (91.2)	463 (92.6)	0.350
Is the injection skin site cleansed and disinfected before the injection?	234 (93.6)	238 (95.2)	472 (94.4)	0.560
Is there an available separate clean area for preparing medications?	223 (89.2)	208 (83.2)	431 (86.2)	0.034*
Are all items for safe injection prepared on a clean tray?	235 (94.0)	198 (79.2)	433 (86.6)	0.001*
Does the injector use sterile and closed syringe/IV cannula?	243 (97.2)	245 (98)	488 (97.6)	0.390
Using ready-made sterile water or manufacturer’s water for dilution of drugs	242 (96.8)	241 (96.4)	483 (96.6)	0.500
Make sure of expire and validity of drugs as exposure to heat or light	217 (86.8)	244 (97.6)	461 (92.2)	0.001*
Disinfection of the medication self-sealed rubber cap especially the multiple dose ones	202 (80.8)	208 (83.2)	410 (83.0)	0.280
Disinfectants are stored and refilled in a correct method	230 (92)	244 (97.6)	474 (94.8)	0.008*
There is complete separation between storage and patient care areas	242 (96.8)	242 (96.8)	484 (96.8)	0.660
Assure fixation of the IV cannula site	245 (98.0)	219 (87.6)	464 (92.8)	0.001*
Apply aseptic non touch technique	238 (95.2)	206 (82.4)	444 (88.8)	0.001*
Medical plaster is not contaminated (dealing with it so as not to contaminate)	218 (87.2)	225 (90)	443 (88.6)	0.199
A gauze barrier is used when breaking glass ampoules to prevent stick injury	202 (80.8)	205 (82.0)	407 (81.4)	0.409
Clean, disposable, single-use gloves are used for each patient	230 (92.0)	166 (66.4)	396 (79.2)	0.001*
Hand washing or alcohol hand rub is used before wearing gloves	195 (78.0)	230 (92)	425 (85.0)	0.001*
Preparing patient’s skin is done in an appropriate aseptic method	244 (97.6)	217 (86.8)	461 (92.2)	0.001*
No touch of the injected vein after preparation (asepsis)	242 (96.8)	235 (94)	477 (95.4)	0.100

*R* items that are negatively worded and right response is NO

*P value ≤ 0.05 is considered significant

Comparing the 2 hospitals as regards the safe injection practices score revealed an almost equal mean total score of 27.39 ± 2.17 and 27.13 ± 3.11 respectively with no significant difference (Table 5).

**Table 5:** Comparison of safe injection practice score among the study groups

Score	Hospital			*P* value
	Qassim Hospital *N* (%)	Beni-Suef UH *N* (%)	Total	
Poor practice (score 0–10)	2 (0.8)	2(0.8)	4 (0.8)	0.146
Fair practice (score 11–20)	4 (1.6)	3(1.2)	7 (1.4)	
Good practice (score 21–31)	244 (97.6)	245 (98)	489 (97.8)	
Mean ± SD of total score (total score = 31)	27.13 ± 3.11	27.39 ± 2.17	27.27 ± 2.68	

Exposure to needle stick injury and needle disposal showed a significant difference between the two hospitals in favor of less exposure to NSI among MCH-Qassim nurses (5.6%) compared to the 32.4% among BSUH group of nurses, while only 75% of the MCH-Qassim nurses reported disposing the needles as a one unit compared to the 95% for the BSUH group of nurses reflecting better disposition of sharps/needles among the Egyptian group of study nurses (*P* = 0.001) (Table 6).

**Table 6:** Exposure to needlestick injuries (NSIs) and methods of needle disposal

Part III—4 items	Hospital			*P* value
	Qassim Hospital *N* (%)	Beni-Suef UH *N* (%)	Total	
Have you been exposed to NSIs in the past year?				
No	236 (94.4)	169 (67.6)	405 (81)	0.001*
Yes	14 (5.6)	81 (32.4)	95 (19)	
How many times have you been exposed to NSIs?				
Never	238 (95.2)	166 (66.4)	404 (80.8)	0.001*
1 time	12 (4.8)	53(21)	65 (13)	
2 times	0 (0)	26 (10.4)	26 (5.2)	
3 times	0 (0)	4 (1.6)	4 (0.8)	
4 times	0 (0)	2 (0.8)	2 (0.4)	
Have you been exposed to other sharps injuries?				
Never	238 (95.2)	111 (44.4)	349 (69.8)	0.001*
Surgical scalpel	1 (0.4)	41 (16.4)	42 (8.4)	
Broken medication vials	7 (2.8)	11 (4.4)	18 (3.6)	
Needle of cannula	4 (1.6)	87 (34.8)	91 (18.2)	
Needles are disposed by				
As one unit	188 (75.2)	238 (95.2)	426 (85.2)	0.001*
Bend and disposal	7 (2.8)	12 (4.8)	19 (3.8)	
Separate needle and disposal	48 (19.2)	0 (0)	48 (9.6)	
Leave syringe with patient	2 (0.8)	0 (0)	2 (0.8)	
Two-hand recapping and disposal in sharps box	4 (1.6)	0 (0)	4 (0.8)	
Two-hand recapping and disposal in non-medical waste	1 (0.4)	0 (0)	1 (0.4)	

*P value ≤ 0.05 is considered significant

## Discussion

The present study revealed high almost similar percentages (93.2–98%) of an implemented safe injection, infection control, and post-exposure needle stick injury policies and procedures in both hospitals, reflecting appropriate awareness as regards safe injection practice. These results are contrary to the results reported by a similar study in Gharbia, Egypt [11], and by an African study reporting that 62.1% of the respondents reported that such policies are not in existence in their hospitals [12].

In addition, higher safe injection (94.8%) and waste sorting (84.8%) training percentages were reported among the BSUH nursing staff compared to the MCH-Qassim training (70% for each category), reflecting a higher mean safe injection awareness score among the Egyptian participants**.** The present findings are similar to the reported 91% safe injection practice training in a Romanian study [13] and higher than that reported (27–33%) in Nepal, Ethiopia, and Bangladesh [14–16], reflecting the national interest of safe injection training practice among the current study hospitals.

Similarly, high awareness was reported among nurses in both study hospitals as regards measures to be taken after NSI (96% and 92.8%) and blood-borne diseases transmitted by unsafe injection practice (100% and 97.9%). These findings are similar to the reported 92.1% awareness of nurses for measures to be taken after NSI in West Bengal [17], higher than the reported 68% in Patiala, North India [18], and similar to the reported high percentage of awareness of blood-borne diseases transmitted by unsafe injection practice in India [19], West Bengal [17], and Nigeria [20].

Complete vaccination doses against hepatitis B among the 2 hospitals’ nursing study group showed a lower percentage among the MCH-Qassim (62.4%) participants compared to the BSUH (76%) participants. Higher rates were reported by other studies conducted in Nepal (76.8%) [14], Pokhara, Iran (82.3%) [21], Rawalpindi (82.7%) [22], and Karachi (73%) [23]. On the contrary, the vaccination coverage for both hospital groups of nurses was higher than that reported in two similar studies conducted in (52.2 and 21.1% .2% respectively) [24, 25]. Health care providers must be provided with full vaccination coverage for patients’ self-safety.

In this study, good awareness towards safe injection (92% and 99.2%) of the MCH-Qassim and BSUH nursing staff were reported. These rates are similar to the reported 90% good knowledge in a Nigerian study [26] but contrary to the low (37.7%) awareness score reported in Asaba, Nigeria [27], and the rate reported in Esan central LGA of Edo State, Nigeria [28].

Injection safety practices in both hospitals were satisfactory. Most of the observed nurses (95.6%) used new syringes and needles for each injection. These findings were consistent with the reported 95.7% new syringe and needle usage in a similar Egyptian study [11] and the 100% new syringe usage in a Romanian study [13] and higher than the reported 72% in another study in Nepal [14].

Safe injection practice was observed among nurses in this study with a minor difference between the 2 hospital groups. For the item *safe injections are prepared on a clean tray*, 94% and 79.2% of the observed nurses among MCH-Qassim and BSUH showed a correct practice respectively. A finding which is higher than a similar study conducted in SSKM Hospital, Kolkata, and West Bengal reported that 60% of the nursing personnel maintained the use of clean tray for safe injections [25].

As for the item *washing hand or using alcohol hand rub before wearing gloves*, 78% and 92% of the MCH-Qassim and the BSUH nursing staff were observed to perform a correct hand hygiene practice, percentages which are lower than the reported percentages in similar Nigerian (78.7%) [20] and Nepalese (63.2%) studies [14] and higher than the lower percentages reported in different studies for hand wash practices: 20% in Nigeria [29], 20% in Patiala [18], 12.5% in West Bengal [25], 4.6% in India [30], and 3.6% in a similar Egyptian study [11]. The discrepancy may be attributed to the higher international awareness and practice training for the hand hygiene practice in the recent years.

Of note, for the item *needles separated from its syringe inside sharps disposal box*, there was a statistically significant difference between the 2 hospital nursing groups, where 97.6% of needles were found not separated inside the sharps box in BSUH vs. 92.8% for the MCH-Qassim. In addition, for the item presence of sharps disposal box near patient care areas, it was observed to be present in 97.6% in BSUH and 93.6% in MCH-Qassim (*P* = 0.001). These findings reflect a better practice than that reported in a similar Nigerian study which revealed that 25% of nurses frequently leave sharps at the patient’s bedside [12].

Preparing patient’s skin in an appropriate aseptic method was higher in MCH-Qassim (97.6%) than in BSUH (86.8%). Similarly, 99.03% of injections were prepared clean using aseptic precautions in a study conducted in India [19].

In the current study, a high score of safe injection practice among nurses of the 2 study hospitals was reported (97.6% and 98%). This finding is contrary to the reported low percentage (40 to 61.4% from North to West India) of safe injection practice [31, 32]. The good awareness and safe injection practices among the 2 hospital study nurses in the present study may be due to implementation of universal standard infection control practices in the Egyptian and Saudi Arabian nursing curriculum and the continuous professional training programs.

Nurses who have experienced needle stick injury in the past 12 months before the study constituted 5.6% among the MCH-Qassim nurses compared to 32.4% among the BSUH group of nurses. These findings are lower than that reported in a Nigerian study with a NSI of 15.8% [20] when compared with the MCH-Qassim group of nurses while higher for the BSUH-Egypt ones. In addition, NSI among both study groups are far less than that reported in other studies: 67.6% and 40.4%, in the 2 hospitals in Ibadan, Nigeria [12], and 50% in India [33]. This disagreement might reflect the national interest in both countries towards safe injection training practice among the current study hospitals and positive attitude and appropriate implementation of safe injection policy and procedures.

In this study, the practice of two-handed recapping of needles after use and disposal in sharps box or disposal in non-medical waste constituted very low percentage in both hospitals (0.8% and 0.4%, respectively). Findings which are far below than that reported (71.4%) in a similar Egyptian series [11] and in other developing countries reported percentages of 9.05 to 17% in two Indian studies [19, 34], 11% in Pakistan [35], 19.1% in Nigeria [20], and 58% in Cambodia [36].

### Limitations of the study

It is a single hospital questionnaire-based observational study in each country aimed to describe the pattern of safe injection practices among nurses. A single observation might not reflect the regular practice of nurses. There is a possibility of bias as healthcare providers may alter their behavior when they know that they are being observed. The possibility of bias is very high in the reported needlestick injuries; the main reasons stated for not reporting were time constraint and low perceived risk of disease transmission due to incident. This study did not aim for the consequent management of NSIs for the participants.

## Conclusions and recommendations

Awareness and practice of injection safety were found to be good among nurses of BSUH, Egypt, and MCH, Qassim, Saudi Arabian. Appropriate training courses were implemented for the observed group of nurses. Continuous health care professional development for infection control and safe injection practice should be encouraged. Early preventive intervention and reporting of NSIs to hospital administration should also be an essential part of injection safety. Frequent infection control auditing is mandatory to ensure safe injection practices.

## Data Availability

All data generated or analyzed during this study are included in this published article.

## Abbreviations

BSUH: Beni-Suef University Hospital
IV: Intravenous
MCH: Maternal and Child Hospital
NSIs: Needlestick injuries
SPSS: Statistical Package for Social Science
