# Correction to: Safe injection awareness and practices among nursing staff in an Egyptian and a Saudi hospital

**DOI:** 10.1186/s42506-020-00038-x

**Published:** 2020-02-25

**Authors:** Manal M. Anwar, Alshimaa M. Mohamed Lotfy, Afaf A. Alrashidy

**Affiliations:** 1 Department of Public Health and Community Medicine, Faculty of Medicine, Beni-Suef University, Beni Suef, Egypt; 2 Ministry of Health, Al Qassim Region, Kingdom of Saudi Arabia

**Correction to: J Egypt Public Health Assoc**

**https://doi.org/10.1186/s42506-019-0018-5**

Following publication of the original article [1], we have been notified that one of the authors’ given names is not reflected correctly.

Author name now is Alshimaa A. Mohamed Lotfy, it should be Alshimaa M. Mohamed Lotfy.
